# Inflammatory Myofibroblastic Tumor of the Lung and the Maxillary Region: A Benign Lesion with Aggressive Behavior

**DOI:** 10.1155/2013/879792

**Published:** 2013-03-06

**Authors:** Lorena Gallego, Tania R. Santamarta, Verónica Blanco, Luis García-Consuegra, Tommaso Cutilli, Luis Junquera

**Affiliations:** ^1^Department of Oral and Maxillofacial Surgery, Cabueñes Hospital, 33394 Gijón, Spain; ^2^Department of Oral and Maxillofacial Surgery, Central University Hospital, 33306 Oviedo, Spain; ^3^Department of Pathology, Central University Hospital, 33306 Oviedo, Spain; ^4^Italy Health Sciences Department, Maxillofacial Surgery Unit, University of L'Aquila, 67100, Italy; ^5^Oral and Maxillofacial Surgery, Dental School, University of Oviedo, Catedrático José Serrano Street, 33009 Oviedo, Spain

## Abstract

Inflammatory myofibroblastic tumor (IMT) is a rare mass-forming lesion characterized by fibroblastic or myofibroblastic spindle cell proliferation with varying degrees of inflammatory cell infiltration. Although it has been reported in virtually every organ in the body, the lung is the most common site of involvement. Extrapulmonary IMTs, although rare, have been reported and are characterized by different, more aggressive behavior. We report an extremely rare case of maxillary metastases of pulmonary IMT. Lung IMT was initially misdiagnosed, and oral lesion mimicked clinically and radiologically a radicular cyst. On histologic examination, cells exhibited diffuse and intense immunoreactivity for **α**-smooth muscle actin and vimentin whereas both pulmonary and oral IMTs presented absence of cellular atypia and lack of expressivity of oncogenic determinants. Distant metastases of lung IMT are extremely unusual, and this is the first report to our knowledge with this particular clinical course. Despite the possibility that the present case could also represent a metachronous multifocal IMT, with pulmonary and extrapulmonary lesions, similar histopathological and immunohistochemical patterns in lung and maxillary region suggest a metastatic course.

## 1. Introduction

Inflammatory myofibroblastic tumor (IMT) is a rare massforming lesion characterized by fibroblastic or myofibroblastic spindle cell proliferation with varying degrees of inflammatory cell infiltration. Because of these unknown factors, IMT was referred to by several different terms until the World Health Organization (WHO) classified IMT as a distinct entity in 1994 [1]. IMTs belong to the group of soft tissue tumours and have also been named plasma cell granuloma, inflammatory pseudotumour, xanthoma xanthomous pseudotumour, fibrous histiocytoma or histiocytoma, mast cell tumour or mast cell granuloma. The majority of IMT lesions are present in the lungs. Extrapulmonary IMTs, although rare, have also been reported and are characterized by different, more aggressive behavior. The frequency of upper respiratory tract accounts for 11% of extrapulmonary cases and the remainder of the head and neck sites, less than 5% [2].

We report an extremely rare case of maxillary metastases of pulmonary IMT. Distant metastases of lung IMT are extremely unusual, and this is the first report to our knowledge with this particular clinical course. Histological and immunohistological findings are analyzed. 

## 2. Case Report

In February 2011, a 53-year-old female patient was referred to our Department of Oral and Maxillofacial Surgery with a chief complaint of swelling and pain of the left upper maxillary region. The symptoms were noticed 2 months previously, after root canal treatment of upper left canine. There was no medical history of note but a remarkable history of pneumonia surgically treated two years ago.

**Figure 1:**
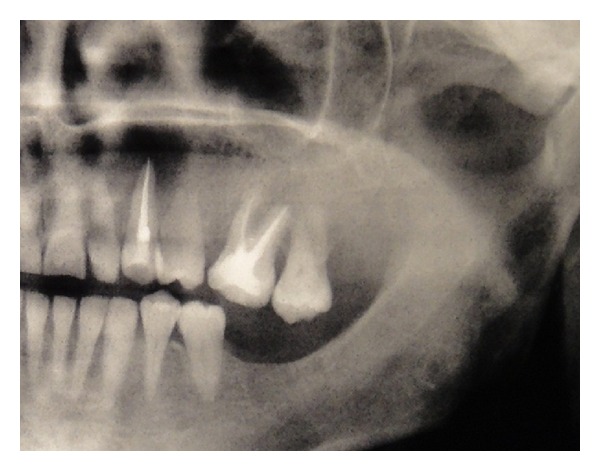
Panoramic radiograph revealing a radiolucency of about 2 cm of diameter in the periapical region of the endodontically treated left canine.

Extraorally, the swelling was diffuse, nontender, with increased local surface temperature and redness in the left infraorbital region. Intraorally, no lesions were observed. There was noted pain on percussion of the endodontically treated canine and adjacent premolar teeth. Radiograph showed root canal filling extending 1 mm beyond the canine root apex and imprecise periapical radiolucency with a diameter of about 2 cm (Figure 1). No root resorption was detected. Previous radiographs nine months before root canal treatment showed no clear radiolucency. Then, a provisional diagnosis of radicular cyst was considered, and a decision was made to perform periapical surgery under local anaesthesia. 

**Figure 2:**
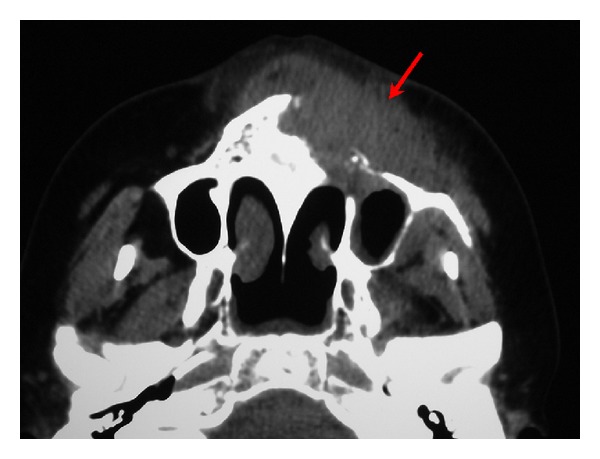
Axial CT image showing an infiltrative mass in the left maxillary region with destruction of the maxillary bone anteriorly wall, invading skin.

Intraoperatively, a cyst-like lesion with unclear margins occupied with globular soft tissue was discovered. All surgical material was sent for histopathologic examination. A postoperative CT showed an infiltrative lesion in the left maxillary sinus with destruction of the maxillary bone anteriorly and minimal destruction of the infraorbital wall, invading skin (Figure 2). 

Although the histopathologic examination was consistent with an active and chronic inflammation without atypical mitosis, a lymphoproliferative or malignant tumor was suspected due to the CT images. A skin and subcutaneous tissue biopsy was performed. Histopathology examination revealed again inflammatory changes without a definitive diagnosis of the lesion. 

**Figure 3:**
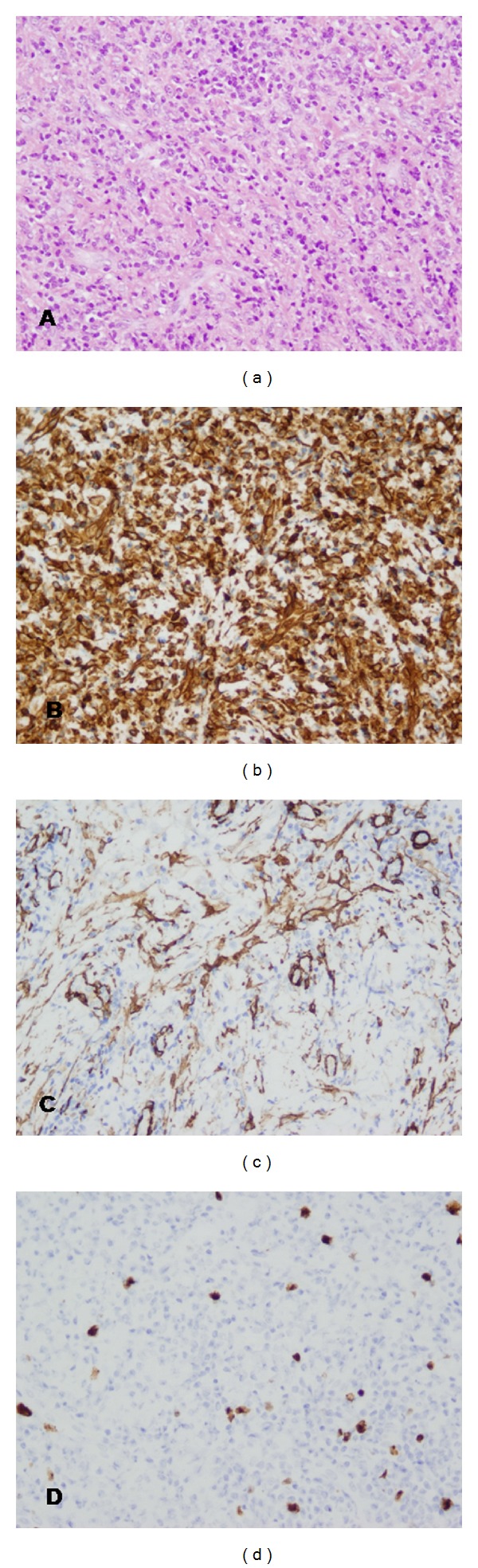
Histological and immunohistochemical examination of the maxillary mass. (a) Hematoxylin-eosin staining demonstrating spindle cells sprinkled with inflammatory cells, with a predominance of plasma cells and lymphocytes (original magnification ×200). (b) immunohistochemical staining showing strong reactivity for vimentin (original magnification ×200). (c) Immunohistochemical staining showing moderate reactivity for *α*-smooth muscle actin (original magnification ×200). (d) Immunohistochemical staining showing focal and weak immunoreactivity of the tumor cells for Ki-67 (original magnification ×200).

Then, the patient underwent a total excision of the mass under general anaesthesia in April 2011. Postoperative histopathology of the surgically excised material revealed a solid, nonencapsulated mass, which is composed of compact spindle cells sprinkled with inflammatory cells, with a predominance of plasma cells and lymphocytes (Figure 3(a)). Special stains for microorganisms, including mycobacteria, fungi, and virus, were negative. The neoplastic cells showed myofibroblastic phenotype and reacted immunologically with vimentin and alpha-smooth muscle actin (Figures 3(b) and 3(c)), while a smaller number was positive for CD5, CD20, and Ki67 (Figure 3(d)). Staining for desmin, CD117, p53, and S100 was negative. With these findings, the diagnosis of IMT was made.

**Figure 4:**
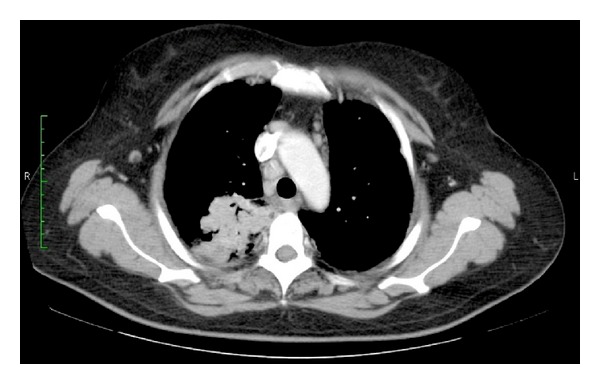
Computed tomography (CT) scan showing an irregular mass in the right lung field. This lesion was initially diagnosed as a cryptogenic organizing pneumonia.

As referred, the patient had a remarkable history of pneumonia surgically treated in January 2009. Histology of lung demonstrated intra-alveolar inflammatory cell infiltration and prominent lymphoid infiltrate with obliteration of bronchiolar space. At that time, the diagnosis based on computed tomography (CT) and histopathology study of surgical specimen was cryptogenic organizing pneumonia (Figure 4). Corticosteroid and calcifediol therapy was prescribed for ten-month duration. During the steroid therapy, the symptoms disappeared and a marked shrinkage of the mass and radiographic improvement was achieved. Prednisolone was gradually tapered and finally discontinued in February 2010, and no regrowth of the tumor was observed. After treatment, CT scans of the chest showed no significant changes in lesion size. The patient was still controlled by Respiratory Care Department.

**Figure 5:**
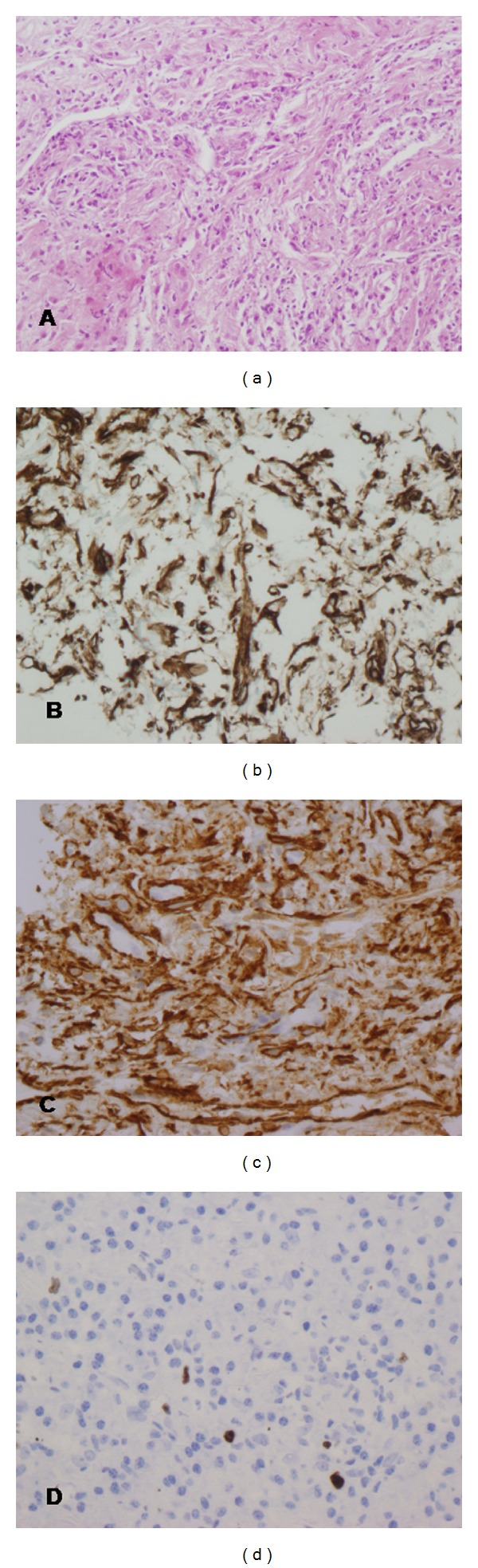
Histological and immunohistochemical examination of the pulmonary mass. (a) Hematoxylin-eosin staining demonstrating spindle cells sprinkled, with a predominance of plasma cells and lymphocytes (original magnification ×200). (b) immunohistochemical staining showing strong reactivity for vimentin (original magnification ×400). (c) Immunohistochemical staining showing strong reactivity for *α*-smooth muscle actin (original magnification ×400). (d) Immunohistochemical staining showing weak immunoreactivity of the tumor cells for Ki-67 (original magnification ×400).

In view of such findings in maxillary region, histopathological results of the previous lung lesion, initially suggesting a cryptogenic organizing pneumonia, were carefully revised. A complete immunohistochemical staining was performed to rule out a possible metastases of previous misdiagnosed lung IMF. In fact, inflammatory cells, with a predominance of plasma cells and lymphocytes (Figure 5(a)), reacted immunologically exactly the same as maxillary IMT. Lung tumor cells reacted positive with vimentin and alpha-smooth muscle actin (Figures 5(b) and 3(c)), smaller number was positive for Ki67 (Figure 5(d)), and staining for desmin, CD117, p53 and S100 was negative. Thus, considering these findings, the specimen was diagnosed as lung IMT with metastasis to the maxillary region. 

The patient's recovery was uneventful. At her most recent followup, 20 months after maxillary resection, there was no sign of oral recurrence. The pulmonary IMT is carefully reviewed with no regrowth or chest symptoms observed to date.

## 3. Discussion

IMT was first reported in 1905 by Birch-Hirschfield, but its etiology remains unknown. In 1994, the World Health Organization defined IMT as an intermediate soft tissue tumor that is composed of myofibroblast-differentiated spindle cells and accompanied by numerous inflammatory cells, plasma cells, and/or lymphocytes [1]. The term pseudotumor was coined because these lesions mimic expansive, invasive malignant tumors, both clinically and radiologically. Recently, the concept of IMT being a benign reactive lesion has been challenged owing to the clinical demonstration of recurrences as high as 37%, cytogenetic evidence of acquired clonal chromosomal abnormality, and the presence of regional metastases [3–5]. The term inflammatory fibrosarcoma (IFS) is used to describe the most aggressive varieties with documented metastasis [6].

Although it has been reported in virtually every organ and site in the body, the lung is the most common site of involvement. When the tumor arises in the lungs it appears as a nodular lesion, usually with completely benign behavior. However, the occurrence of the tumor at extrapulmonary sites, histologically the same, may be accompanied with some special aggressive characteristics and then classified as IFS [7]. It is worth mentioning that the WHO does not classify IFS as a single entity; however, based on the study by Meis and Enzinger [4], several authors share the opinion that there is a difference between these 2 tumors (focused on the metastatic potential of each).

Pseudotumours are relatively rare in the head and neck, excluding the orbit and cerebrum. IMT has been reported to occur in the larynx, orbit, maxillary sinus, mandible, mouth, parapharyngeal space, tonsils, thyroid, parotid, and lacrimal glands [8]. Associated clinical findings include fever, tenderness, and erythema over the affected region. The imaging findings of IMT are a mildly enhancing soft-tissue mass without any internal calcification or bone destruction revealed by contrast-enhanced CT, although in our case, the tumor had infiltrated all the bony walls of the involved sinus, indicating an aggressive variety of IMT.

Histologically, IMT is composed of inflammatory cells, histiocytes, and fibroblasts. The specific components of IMT can vary and range from those that are dominated by plasma cells to those primarily comprised of myofibroblasts and fibroblasts [9]. The recent WHO classification of soft tissue tumors recognizes 3 basic variants of IMT: (1) loosely organized myofibroblasts in an edematous myxoid background with plasma cells, lymphocytes, eosinophils, and blood vessels, resembling nodular fasciitis; (2) dense aggregates of spindle cells arrayed in a variable myxoid and collagenized background and admixed with a distinctive inflammatory infiltrate, diffuse clusters of plasma cells, and lymphoid nodules, resembling fibrous histiocytoma or fibromatosis; and (3) collagen sheets with scattered plasma cells and eosinophils resembling a scar or desmoid tumor [10]. Cytologic atypia with nuclear pleomorphism and increased mitotic activity are uncommon features and may be associated with malignant transformation.

We report a case of lung IMT with distant maxillary metastases. This is, to the best of our knowledge, the first report of diagnosis of a maxillary IMT after pulmonary manifestation. Despite the possibility that the present case could also represent a metachronous multifocal IMT, with pulmonary and extrapulmonary lesions, similar histopathological and immunohistochemical patterns in lung and maxillary region suggest a metastatic course. With regard to the present case, the supporting histology was more congruent with a diagnosis of IMT than IFS, as evidenced by the absence of frequent mitotic figures, unapparent cellular atypia, and lack of expressivity of several oncogenic determinants, such as p53 and Ki-67. Otherwise, the clinical course of our report suggests biologic aggressiveness, due to the presence of metastases. Those findings disagree with studies that associated potential for aggressive growth, recurrence, and malignant transformation with a high degree of atypia, presence of ganglion-like cells, increased mitotic figures, multinodularity, DNA aneuploidy, elevated Ki-67 proliferative index, and oncogenic protein overexpression, such as ALK, p53, and bcl-2 [11].

A simple excision is the proposed and mainly applied treatment, including postoperative reassessment for at least 10 years. Local recurrence could be related with inadequate resection of the lesion or tumors. The difficulty of completely excising the tumor in most of the cases is related to its location and its vicinity to valuable structures, which by definition are numerous in the head and neck area [12]. Although the number of oral IMTs is limited, seems to exhibit a more favourable clinical course and is distinguished, to date, by the lack of recurrence, malignant transformation, metastasis, and mortality. Metastasis is seen in less than 5% of the cases of IMT [7, 13]. However, bearing in mind the fact that IMT could be multifocal, it is difficult to distinguish between a real metastasis and a synchronous or metachronous primary lesion, as the case reported. Adjuvant radiotherapy has also been reported for the treatment of IMT but because of the small number of reports, it is difficult to adopt safe conclusions.

## 4. Conclusion

In summary, a case of maxillary metastases of pulmonary IMT is described. Lung IMT was initially misdiagnosed, and oral lesion mimicked clinically and radiologically a radicular cyst. On histologic examination, cells exhibited diffuse and intense immunoreactivity for *α*-smooth muscle actin and vimentin whereas both pulmonary and oral IMTs presented absence of cellular atypia and lack of expressivity of oncogenic determinants. This is the first report to our knowledge of an IMT of the lung with oral metastasis.
